# Supplementary material for: The effect of biochar amendments on phenanthrene sorption, desorption and mineralisation in different soils
Source: PeerJ. 2018 Jun 27;6:e5074. doi: 10.7717/peerj.5074 (PMC6026465; doi:10.7717/peerj.5074)

**Supplementary material of “The effect of biochar amendments on ^14^C-labelled phenanthrene sorption, desorption and mineralisation in different soils”, by Moreno Jiménez et al.**

**Table SM1.** Effect of contact time on sorption coefficients of Phe by non-amended and amended soil samples and sand in single component system. Experimental conditions: c_0_ =10 μg Phe g^-1^, amount of soil 100 g L^-1^, 1000-1600 Be ^14^C Phe g^-1^ soil. K_d_ and K_oc_ in non-amended (NoBC) samples (sand, Witten soil, Bottrop soil) and samples amended by 1% (w/w) pine woodchips-derived biochar (PBC), olive pruning-derived biochar (OBC) and rice biochar (RBC).

|  | **Amendment** | **contact time** | | | | | |
| --- | --- | --- | --- | --- | --- | --- | --- |
|  |  | **7 h** | | **24 h** | | **48 h** | |
|  |  | logK_d_ | logK_oc_ | logK_d_ | logK_oc_ | logK_d_ | logK_oc_ |
| **Sand** | NoBc | -0.2 | - | 0.13 | - | 0.10 | - |
|  | PBC | 0.13 | 2.45 | 0.85 | 3.07 | 0.83 | 3.05 |
|  | OBC | 0.74 | 3.11 | 1.79 | 4.16 | 1.80 | 4.15 |
|  | RBC | 0.43 | 2.98 | 1.49 | 4.05 | 1.40 | 4.04 |
| **Witten** | NoBc | 1.18 | 2.85 | 1.33 | 3.70 | 1.34 | 3.68 |
|  | PBC | 1.14 | 2.74 | 1.46 | 3.75 | 1.46 | 3.74 |
|  | OBC | 1.35 | 2.95 | 1.63 | 3.83 | 1.63 | 3.82 |
|  | RBC | 1.23 | 2.85 | 1.53 | 3.86 | 1.53 | 3.85 |
| **Bottrop** | NoBc | 1.71 | 3.29 | 2.11 | 3.89 | 2.10 | 3.88 |
|  | PBC | 1.71 | 3.22 | 1.96 | 3.87 | 1.95 | 3.86 |
|  | OBC | 1.76 | 3.28 | 2.05 | 3.87 | 2.04 | 3.88 |
|  | RBC | 1.67 | 3.21 | 1.93 | 3.87 | 1.93 | 3.89 |

**Figure SM1.** Average K_oc_ (upper graph) and K_d_ (lower graph) in the isotherm of Phe sorption experiment. *The value of K_d_ for sand was -0.12, not visible in the graph. Mean±SE, n=12, grouping all the values of the isotherm for each treatment (1, 2.5, 5 and 10 mg Phe g^-1^ together). Different letters indicate statistical differences (p<0.05) between biochar treatments in each soil/substrate.

**Figure SM2.** Experimental K_d_ or K_oc_ to calculated K_d_ or K_oc_ relationship (right and left side respectively) in treatments where biochars were added to soil. Averages values from the sorption isotherm were used for both parameters. Experimental data were obtained from the isotherm experiment for treatments Witten PBC, OBC and RBC and Bottrop PBC, OBC and RBC. The calculated value for the respective samples was estimated by summing the K_d_/K_oc_ observed in the respective soil system to the K_d_/K_oc_ in each soil (Witten and Bottrop without biochar).


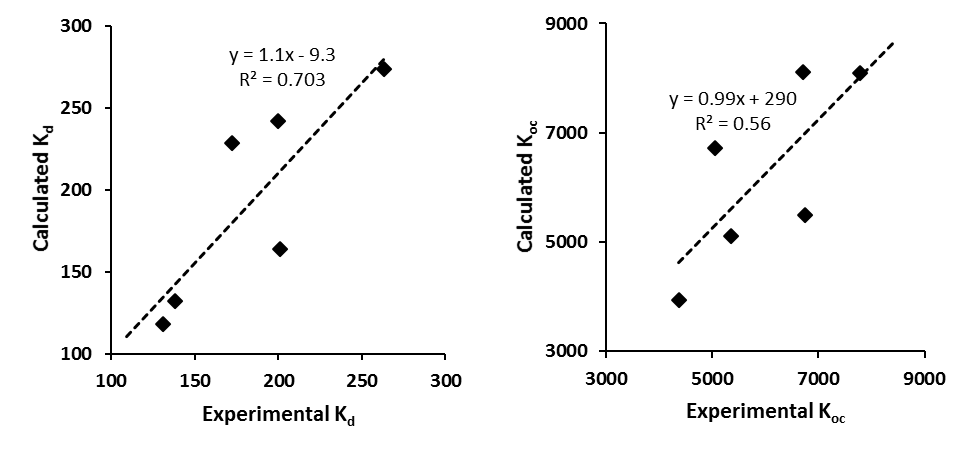

Supplement: Supplemental Information 1 [file peerj-06-5074-s001.docx]
